# Welcome to the New Era: A Completely Wireless Interventional Procedure

**DOI:** 10.7759/cureus.3337

**Published:** 2018-09-19

**Authors:** Jesse T Martin, Paul C Hulsberg, Erik Soule, Michael Shabandi, Jerry Matteo

**Affiliations:** 1 Radiology, Edward Via College of Osteopathic Medicine, Auburn, USA; 2 Interventional Radiology, University of Florida College of Medicine, Jacksonville, USA; 3 Interventional Radiology, University of Florida College of Medicine, Gainesville, USA

**Keywords:** endovascular intervention, interventional radiology, interventional oncology, angiographic embolization, arterial embolization, wireless interventional procedure

## Abstract

The number of minimally invasive interventional radiology (IR) and interventional cardiology vascular procedures performed increases every year. As the number of vascular procedures increases, the need for advanced technology and innovative devices increases as well. Traditionally, as a general rule, a catheter is used in conjunction with a guidewire in such procedures. The underlying principle of IR is to always use a guidewire prior to any advancement of a catheter. This article describes a revolutionary theory that utilizes a new technology and contradicts this basic principle. Using a steerable microcatheter, a bilateral uterine artery embolization was performed from a wrist access with no guidewire. Furthermore, this technique reduced the procedure time by more than half when compared to standard of care. This technique may be applicable to other IR procedures, which could potentially reduce the time critically ill patients spend in the procedure area outside the intensive care unit.

## Introduction

In 1953, Swedish radiologist Sven-Ivar Seldinger developed the Seldinger guidewire catheterization technique, which, with the use of a wire, allows for the placement of a catheter larger than the cannula of the needle used to create the initial vascular access [1]. Charles Dotter further developed this technique in 1964, by performing the first balloon angioplasty, allowing for minimally invasive treatment of atherosclerotic disease. Similarly, Julio Palmaz’s introduction of the balloon-expandable stent in 1985 resulted in a paradigm shift in the treatment of coronary and peripheral artery disease [2]. As demonstrated here, the rotating-tip steerable microcatheter has the potential to transform the field of interventional radiology (IR) by eliminating the need for a guidewire.

The use of a guidewire plays a significant role in maintaining access, aiding in catheter advancement, crossing stenotic lesions, and sub-selecting difficult vessels with the use of specialized curves. It is not uncommon to use multiple types of wires and catheters in a single case. Frequent catheter and wire exchanges make it possible to use one wire/catheter combination for a certain task and switch to a different combination for a subsequent task while maintaining the same access. However, the major drawback of frequent guidewire and catheter exchanges is the associated time consumption.

It is well-established that increased procedure time is associated with increased morbidity [3]. This is especially paramount in the critically ill, or an acutely hemorrhaging patient. Although angiographic therapies often provide life-saving interventions, there is a significant risk associated with transporting these patients from the intensive care unit to the IR suite; thus, minimizing procedure time for these patients is essential [4]. Catheter time, which is inextricably linked to procedure time, carries its own risks. The longer a vessel is catheterized, the more prone it is to spasm or thrombosis. Increased manipulations through wire and catheter exchanges also increase the risk of vessel dissection [5]. These principles are especially true for vessels of smaller caliber. Finally, increased procedural time is associated with increased radiation exposure [6]. While patient exposure is typically not a concern, the cumulative effects of operator exposure are important to recognize and mitigate whenever possible.

With the advent of a steerable microcatheter suitable for vessel selection without a wire, much of the time spent exchanging wires and catheters during procedures could be significantly reduced. The SwiftNINJA steerable microcatheter (Merit Medical Systems, South Jordan, UT, USA) features a 180-degree rotating tip that can be steered by simply turning a plastic dial on the hub of the catheter. This design was originally indicated for the catheterization of difficult and tortuous vessels not amenable to traditional catheterization with a guidewire [7]. Recently, due to increased market availability and decreased costs, its indications have shifted, making it a primary procedural microcatheter rather than a problem-solving device [8].

## Technical report

**Figure 1:**
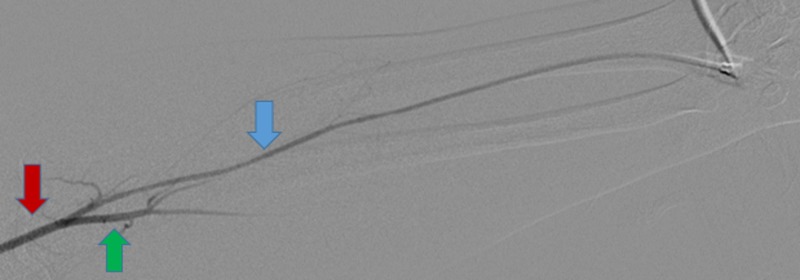
Left radial arteriogram showing normal patency (blue arrow) and brachial artery (red arrow) with contrast reflux partial filling the ulnar artery (green arrow).

After 2% lidocaine was infiltrated into the subcutaneous and deeper tissues of the left wrist, access to the left radial artery was performed using real-time ultrasound guidance. A 5 French hydrophilic-coated introducer sheath (Terumo Glidesheath Slender, Somerset, NJ, USA) was introduced. The left radial arteriogram was then performed through the sheath which demonstrated normal anatomy with no radial loop (Figure 1).

**Figure 2:**
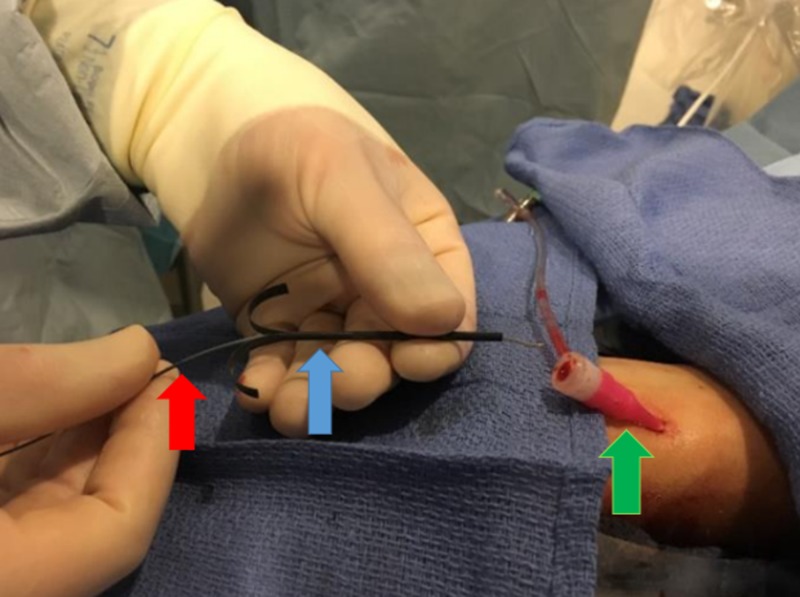
A peel-away introducer (blue arrow) is used to insert the SwiftNINJA steerable microcatheter (red arrow) into the sheath (green arrow).

**Figure 3:**
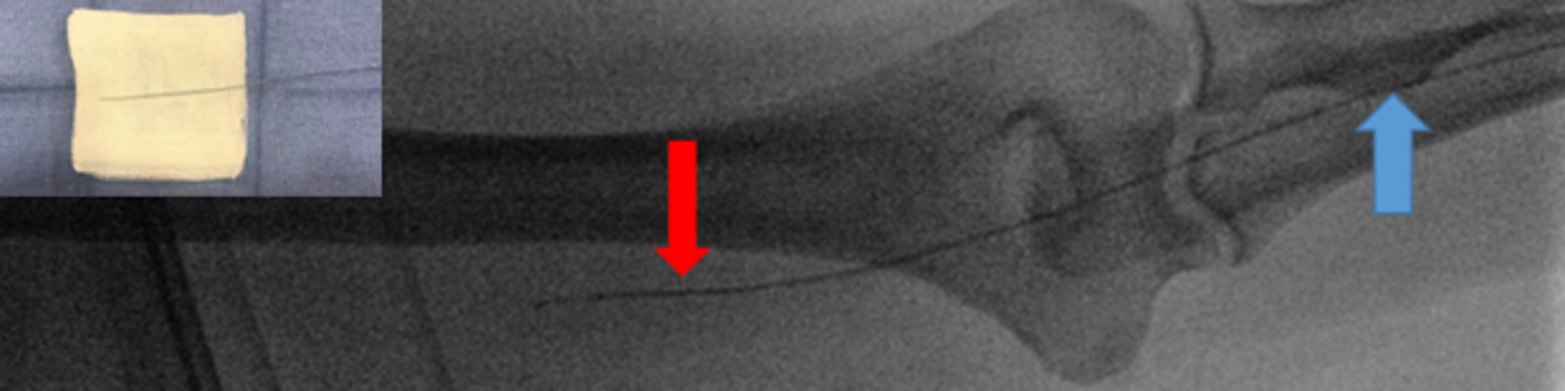
Steerable microcatheter advancing proximally from radial artery (blue arrow) to the brachial artery (red arrow) without a wire. Top left shows a back table image of the catheter in straight position without any controlled torque applied.

**Figure 4:**
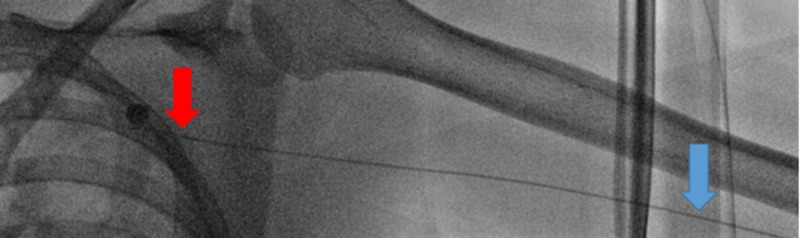
Steerable microcatheter advancing centrally from the brachial artery (blue arrow) to the subclavian artery (red arrow) without a wire.

**Figure 5:**
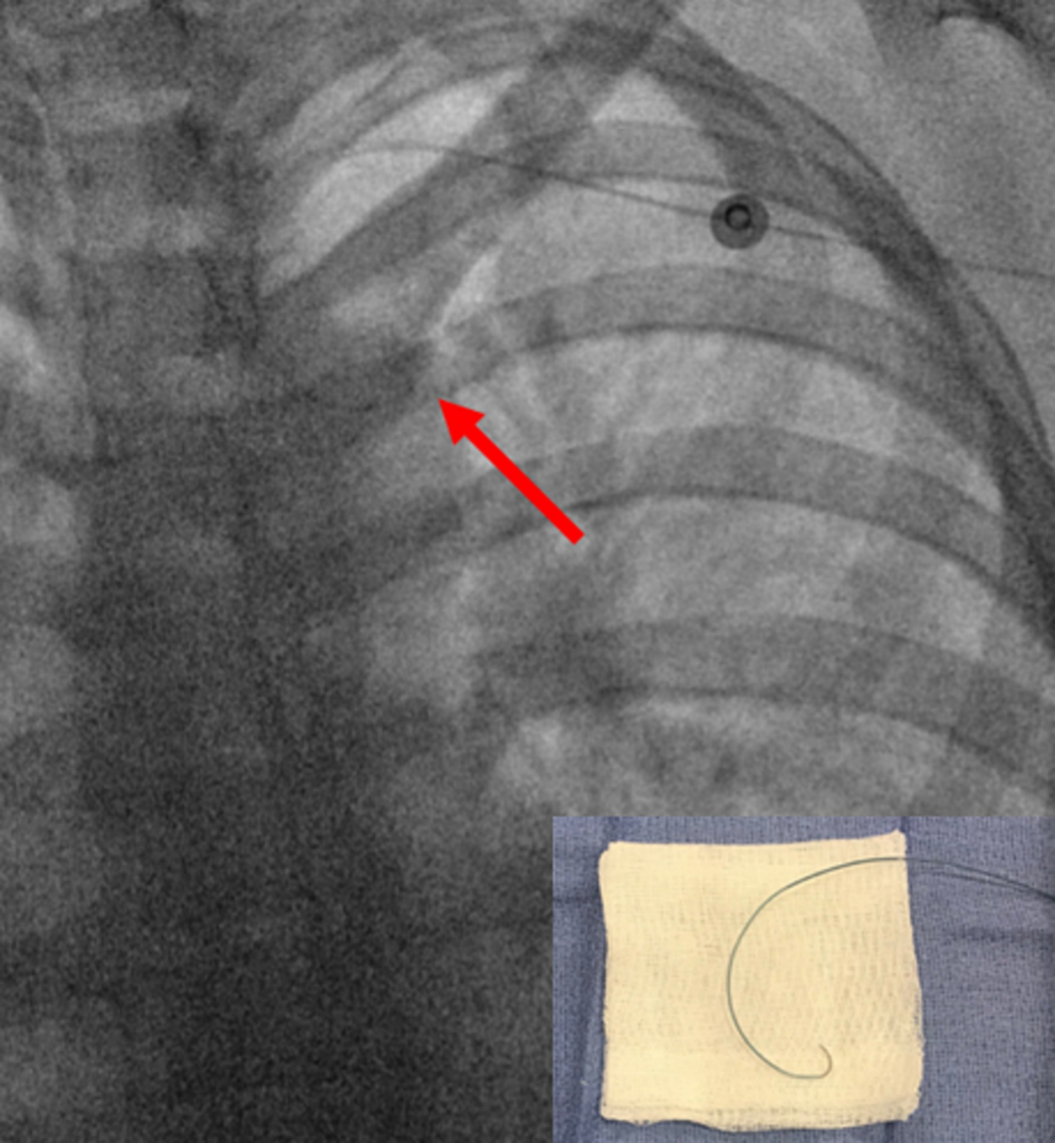
The steerable microcatheter was torqued with the tip (red arrows) pointing into the descending aorta for easy passage around the aortic arch into the descending aorta without a wire. Bottom right shows a back table image of the catheter with maximum torque applied.

Using a peel-away introducer, a 2.4 French 125 cm SwiftNINJA steerable microcatheter (SMC) was advanced directly into the sheath without the use of a base catheter or wire (Figure 2). The SMC was then advanced proximally through the upper extremity radial artery, brachial artery, and subclavian artery (Figures 3-4). The steering dial was used to easily direct the catheter into the descending aorta (Figure 5).

**Figure 6:**
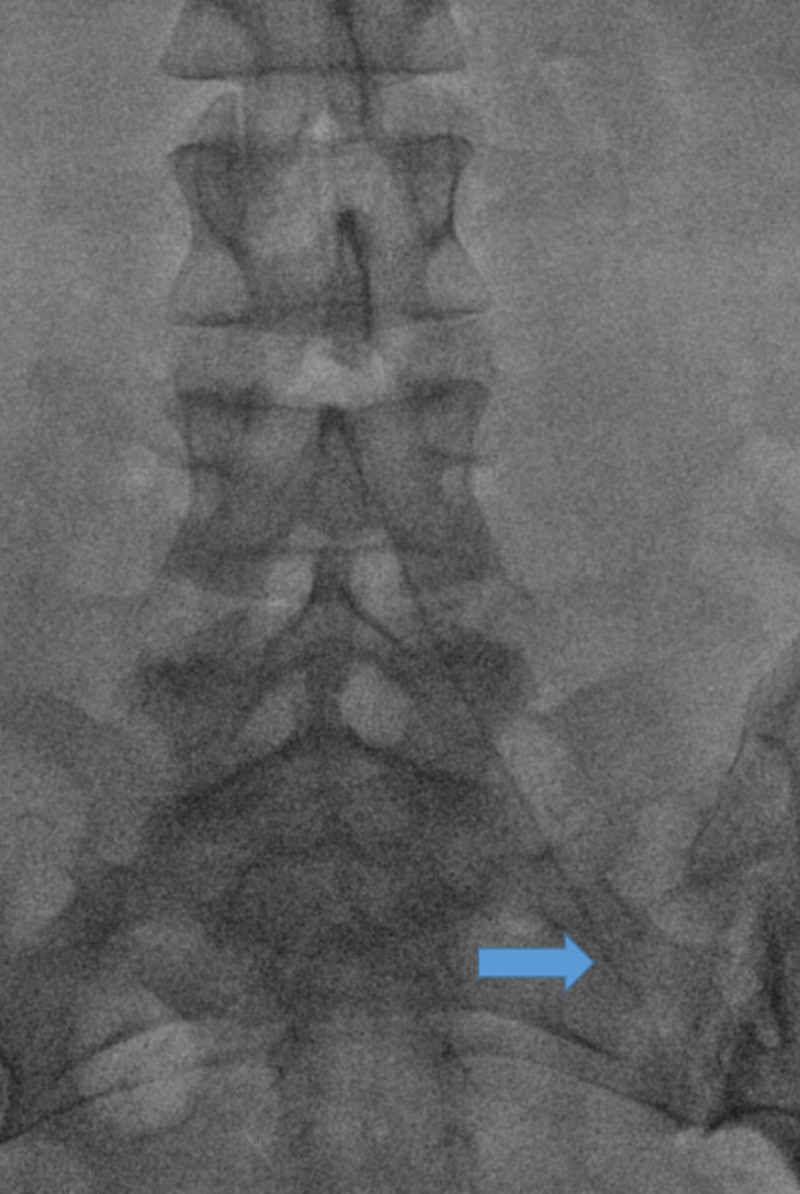
Selective catheterization of the left internal iliac artery with a steerable microcatheter (blue arrow).

**Figure 7:**
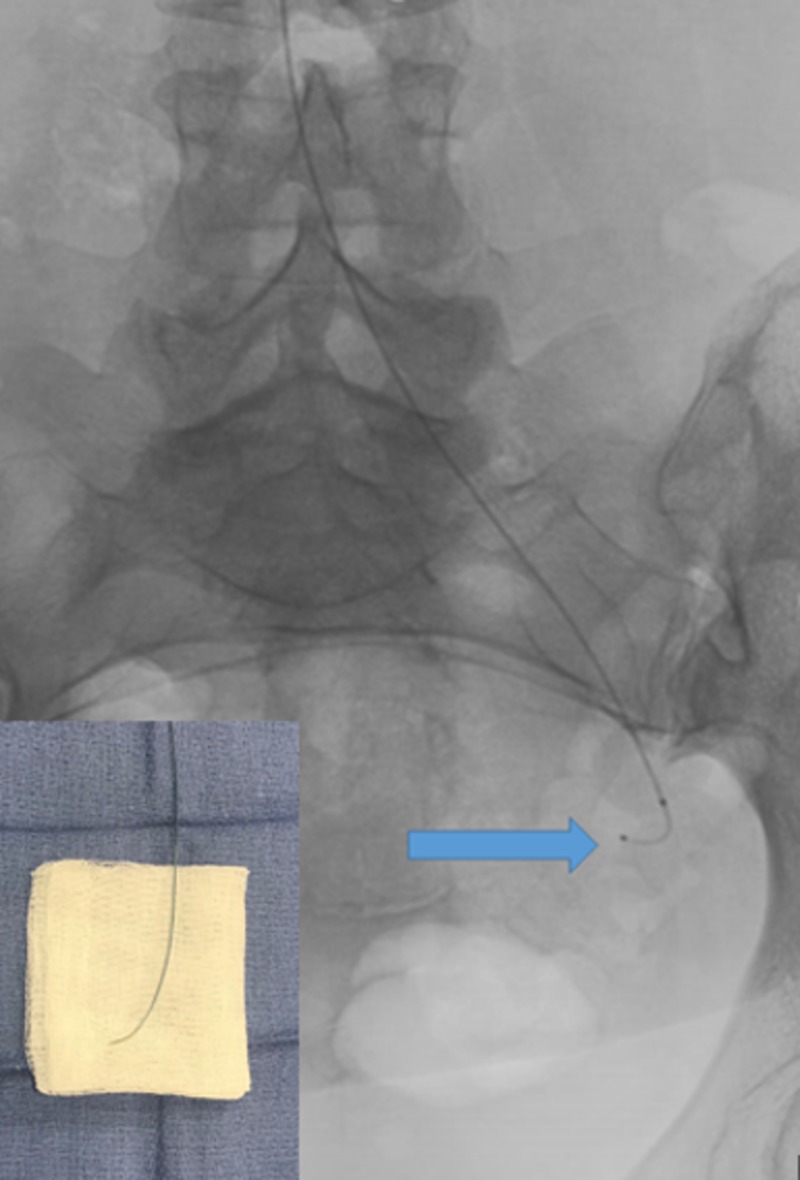
Selective catheterization of the left uterine artery with a steerable microcatheter (blue arrow) without a wire. Bottom left shows a back table image of the catheter with fine torque applied.

**Figure 8:**
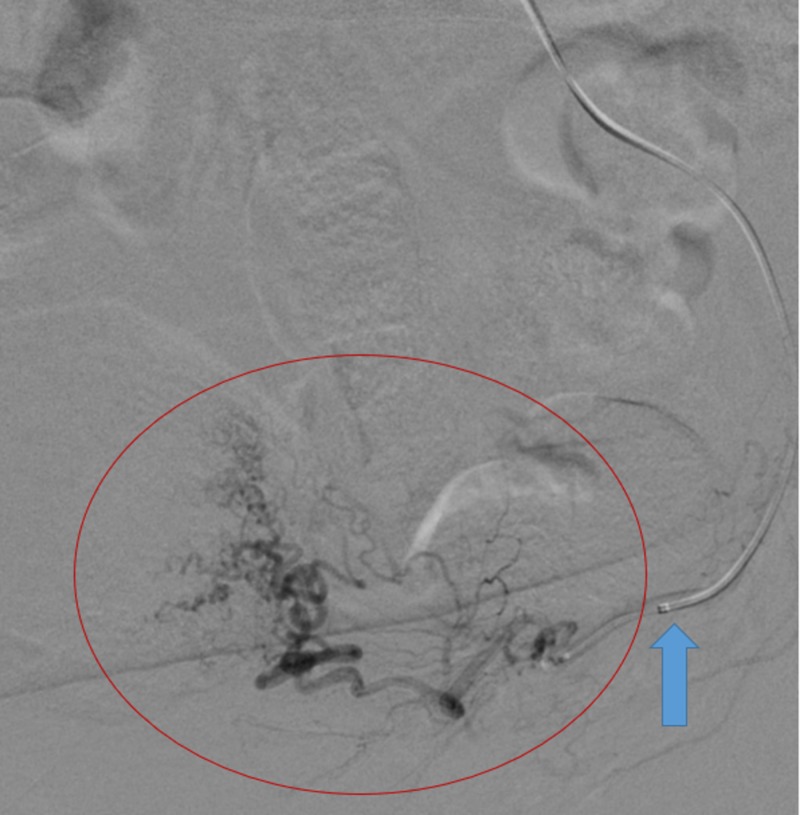
Selective angiography of the left uterine artery (red circle) using the steerable microcatheter (blue arrow) demonstrated marked tortuosity compatible with the history of fibroids.

Using intermittent contrast injections and the steering dial, the microcatheter was then advanced through the left internal iliac artery and down to the distal left uterine artery (Figures 6-7). Selective angiography of the left uterine artery demonstrated marked tortuosity and abnormal vessels compatible with the history of fibroids (Figure 8).

**Figure 9:**
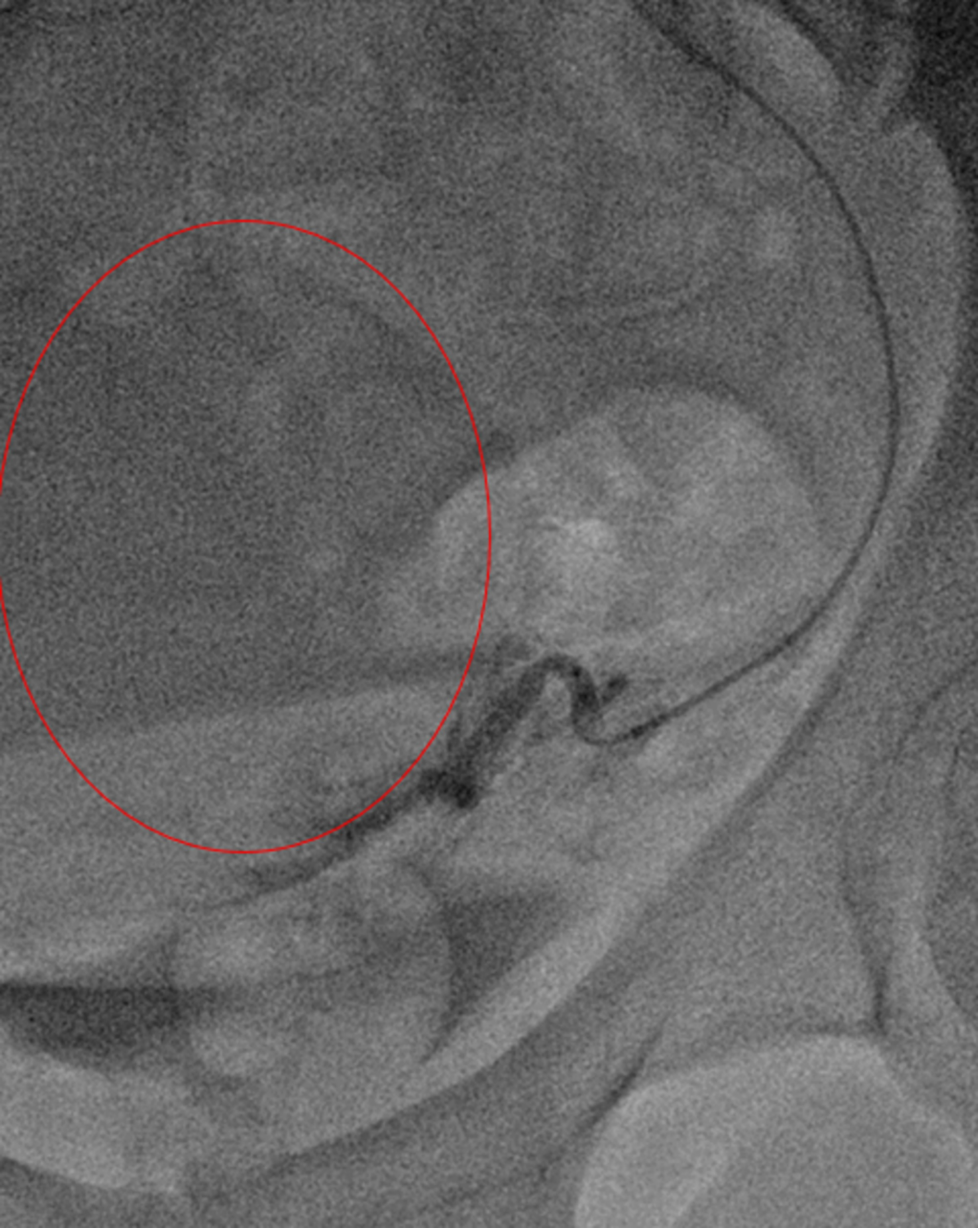
Follow-up post-embolization angiography demonstrated stagnant flow into left uterine artery distribution (red circle).

**Figure 10:**
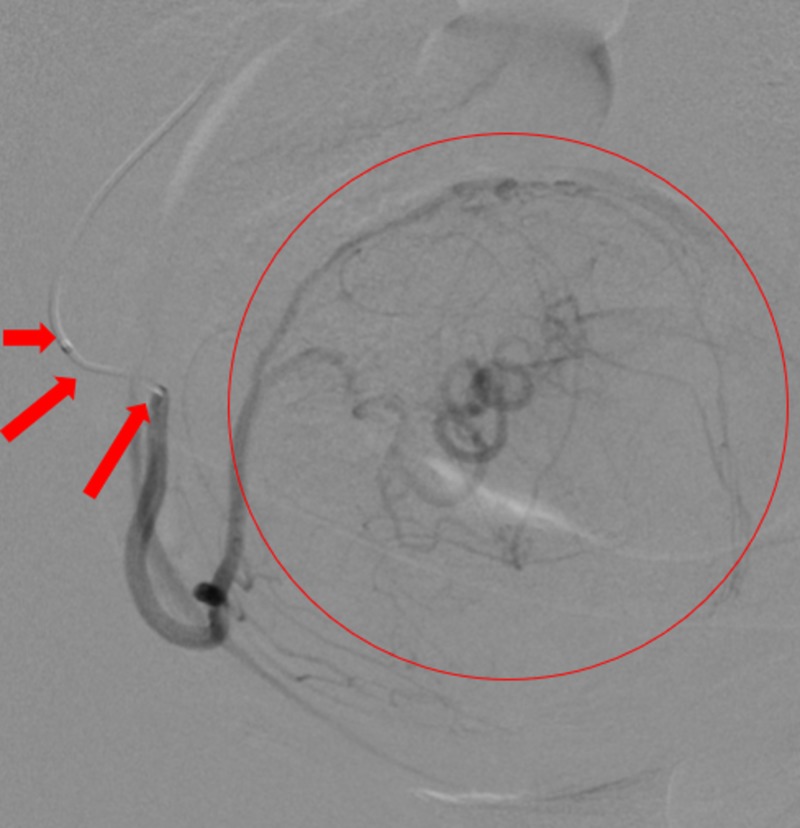
Selective angiography performed by steering the microcatheter (red arrows) into the right uterine artery demonstrated supply of a large fibroid (red circle).

**Figure 11:**
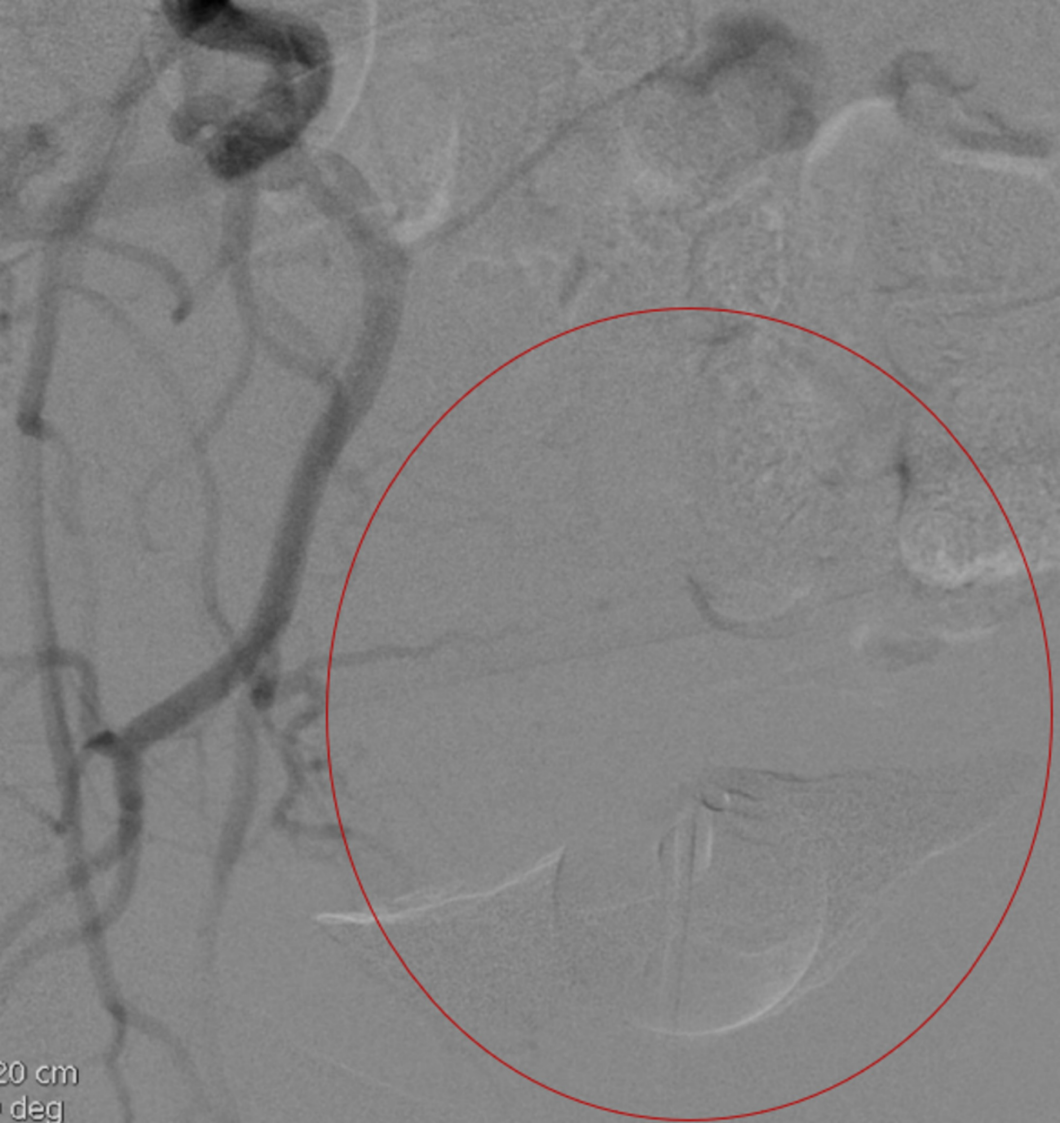
Follow-up post embolization angiography demonstrated lack of flow in the right uterine artery distribution (red circle).

Under fluoroscopic guidance, embolization was performed with 500–710 micron polyvinyl alcohol (PVA) particles mixed with contrast until stasis was observed (Figure 9). The microcatheter was then retracted to the bifurcation and advanced through the right internal iliac artery into the distal right uterine artery. Angiography, embolization, and follow-up angiography were then performed using the same technique on the contralateral side (Figures 10-11). Hemostasis was obtained with the aid of a radial compression band.

## Discussion

With the exception of the microwire used in the radial access set, the entire procedure was performed with a steerable microcatheter without the use of a base catheter, guidewire, or microwire. Despite the use of intermittent contrast injections for vessel selection, the iodinated contrast dose was only 40 mL. This can be attributed to the very small volumes needed for selection, due to the elimination of the need for formal arteriography until the target vessels had already been selected.

The catheter outperformed expectations and was easily advanced up the arm and down the descending aorta by using its steerable tip as described below. Once the catheter tip was visualized within the left common iliac artery, gentle intermittent contrast injections combined with manipulation of the steering handle allowed the left uterine artery to be catheterized without the need for a dedicated internal iliac arteriogram. A follow-up angiography demonstrated extremely slow flow and mild reflux consistent with adequate embolization of bilateral uterine fibroids. The entire procedure was performed successfully within half the time one would typically need using traditional techniques.

By eliminating the need for a base catheter, guidewire, and microwire, this example demonstrates that the wireless SMC technique can effectively decrease the procedure time, catheter time, contrast dose, and radiation exposure. The concept of placing catheters over wires has been the foundation of IR since the advent of the specialty. The utility of the SMC to safely and effectively operate without a guidewire may shift this paradigm. As exemplified in this case, SMCs provide the ability to perform complex procedures without the need for a wire, and in certain situations, without the need for a base catheter. SMCs allow the operator to navigate tortuous vasculature via intermittent contrast injections while simultaneously adjusting the tip in the desired direction and advancing the catheter as necessary. This differs from the conventional method of vessel selection with a wire followed by removal of the wire prior to contrast injection for angiographic runs, which is inefficient and time-consuming. By eliminating numerous wire exchanges and using “real-time road mapping” software, procedure time, as well as radiation and contrast doses, can be reduced [9].

During this procedure, navigation from the left radial artery to the uterine artery was achieved more rapidly than in a typical uterine artery embolization procedure. In fact, the total time between the SMC entering the sheath and its tip reaching the target vessel (left uterine artery) was less than three minutes. Furthermore, the entire procedure was performed in 22 minutes. Comparing this technique with the standard transradial uterine artery embolization (TR-UAE) technique, which has a mean procedure time of 55 minutes, the procedure time was reduced by 60% [10]. Decreased procedure time was accomplished mainly by the lack of guide wire/catheter exchanges, decreased need for road mapping, and ease of catheter navigation. It is clear that the use of an SMC has the potential to decrease procedure time for not only TR-UAE but for most procedures requiring the use of a microcatheter, including technically challenging interventional oncology and neurointerventional cases.

The uterine artery embolization technique showcased in this article is just one of the many procedures that may eventually be performed without a wire. A great number of interventional procedures could benefit from the versatility of the SMC. Indeed, performing a procedure without a wire may very well become the standard for certain IR cases, giving rise to a new era of the wireless interventional procedure.

## Conclusions

Evolution is inevitable. IR is an innovative specialty on the cutting edge of minimally invasive medical procedures. With the advent of new technology, the future is limitless. This report demonstrates a novel technique that challenges the basic principle of interventional vascular procedures that use a guidewire to advance the catheter. This technique was used to safely and effectively perform a uterine artery embolization while decreasing the procedure time by more than half.
